# Tissue factor pathway inhibitor-2 was repressed by CpG hypermethylation through inhibition of KLF6 binding in highly invasive breast cancer cells

**DOI:** 10.1186/1471-2199-8-110

**Published:** 2007-12-03

**Authors:** Hongshen Guo, Yifeng Lin, Hongwei Zhang, Juan Liu, Nong Zhang, Yiming Li, Desheng Kong, Qiqun Tang, Duan Ma

**Affiliations:** 1 Key Laboratory of Molecular Medicine, Ministry of Education, Yixueyuan Road 138^#^, Shanghai Medical College, Fudan University, Shanghai 200032, China; 2 Department of Pathology, Shanghai Medical College, Yixueyuan Road 138^#^, Fudan University, Shanghai 200032, China; 3 Department of Surgery, Zhongshan Hospital, Fenglin Road 180^#^, Fudan University Shanghai 200032, China; 4 Department of endocrinology, Huashan Hospital, Wulumuqi Middle Road 12^#^, Fudan University Shanghai 200032, China

## Abstract

**Background:**

Tissue factor pathway inhibitor-2 (TFPI-2) is a matrix-associated Kunitz inhibitor that inhibits plasmin and trypsin-mediated activation of zymogen matrix metalloproteinases involved in tumor progression, invasion and metastasis. Here, we have investigated the mechanism of DNA methylation on the repression of TFPI-2 in breast cancer cell lines.

**Results:**

We found that both protein and mRNA of TFPI-2 could not be detected in highly invasive breast cancer cell line MDA-MB-435. To further investigate the mechanism of TFPI-2 repression in breast cancer cells, 1.5 Kb TFPI-2 promoter was cloned, and several genetic variations were detected, but the promoter luciferase activities were not affected by the point mutation in the promoter region and the phenomena was further supported by deleted mutation. Scan mutation and informatics analysis identified a potential KLF6 binding site in TFPI-2 promoter. It was revealed, by bisulfite modified sequence, that the CpG island in TFPI-2 promoter region was hypermethylated in MDA-MB-435. Finally, using EMSA and ChIP assay, we demonstrated that the CpG methylation in the binding site of KLF-6 diminished the binding of KLF6 to TFPI-2 promoter.

**Conclusion:**

In this study, we found that the CpG islands in TFPI-2 promoter was hypermethylated in highly invasive breast cancer cell line, and DNA methylation in the entire promoter region caused TFPI-2 repression by inducing inactive chromatin structure and decreasing KLF6 binding to its DNA binding sequence.

## Background

Human tissue factor pathway inhibitor-2 (TFPI-2) is a kunitz-type serine proteinase inhibitor synthesized and secreted into extrocelluar matrix (ECM) by endothelial cells, smooth muscle cells, fibroblasts, keratinocytes and urothelium [1,2]. TFPI-2 readily inhibits trypsin, plasmin, chymotrypsin, cathepsin G, plasma kallikrein and the factor VIIa-tissue factor complex, but not urokinasetype plasminogen activator (uPA), tissue-type plasminogen activator (tPA) or thrombin [3,4]. It had already been reported that the expression of TFPI-2 was down regulated in several invasive tumor cell lines, including choriocarcinoma, glioma, prostate cancer, melanoma and fibrosarcoma, moreover ectopic expression of this gene inhibits tumors growth and metastasis in vivo by regulating pericellular ECM remodeling and angiogenesis [5-10]. Nevertheless, the mechanisms that alter/modify the expression of TFPI-2 gene in cancer cells are not well understood.

Cytosine hypermethylation at CpG dinucleotides in the promoter of tumor suppressor genes represents a major mechanism for gene inactivation in cancer. Methylation at 5' position of cytosine has been reported to alter or interfere with the correct binding of transcription factors to target sequences overlapping CpG dinucleotides [11,12], and it also has a positive effect to recruit methyl-CpG binding activities that associate with histone deacetylases and other chromtin-modifying elements that lead to a transcriptionally silenced state. Many genes are hypermethylated at their CpG islands-containing promoters and subsequently inactivate in human tumors of different etiology [13].

TFPI-2 promoter exhibits typical features of a housekeeping gene with a high GC-rich content (approximately 75%). It has a typical GC box known as binding site for the transcription factor Sp1, and three transcription initiation sites (one major initiation site and the two minor initiation sites, but without canonical TATA and CAAT boxes [8,14]. It also has a potential Kruppel-like factor 6 (KLF6) binding site by bioinformatics. As transcription factors, KLF6 and Sp1 cooperatively transactivate the endoglin promoter of collagen alpha1(I), uPA, TGF-beta1, and TGF-beta receptor type 1 [15-17]. Direct physical interaction between Sp1 and KLF6 was documented by coimmunoprecipitation, pull-down experiments, and the GAL4 one-hybrid system, mapping the KLF6 interaction to the C-terminal domain of Sp1 [15].

Breast cancer is the most common malignancy among females. Hypermethylation of promoter CpG islands, which is frequently observed in breast cancer [18-20], is often associated with transcriptional silencing of the associated gene. In this paper, we explored both genetic and epigenetic mechanisms controlling TFPI-2 expression in human breast cancer cells and the results indicated that TFPI-2 expression could be silenced by promoter hypermethylation by inducing inactive chromatin structure and decreasing KLF6 binding to its DNA binding sequence.

## Results

### Expression of TFPI-2 in breast cancer cells

**Figure 1:**
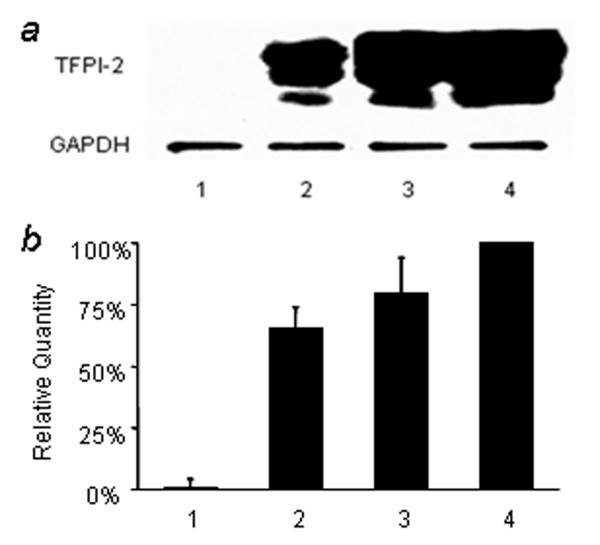
**Expression of TFPI-2 in human breast cancer cells with different metastasis potential**. (A). Western blot analysis of TFPI-2 protein expression. The HUVEC cell line was used as positive control and the protein levels of GAPDH were determined as control. (B). Quantitative real-time PCR analysis of TFPI-2 mRNA levels. All expression levels of TFPI-2 in breast cancer cell lines were normalized to the level of its expression in HUVEC. Lane 1: MDA-MB-435. Lane 2: T47D. Lane 3: MCF-7. Lane 4: HUVEC.

Expression of TFPI-2 protein in human breast cancer cell lines with different metastasis potential was examined by western blotting. As shown in Figure 1a, TFPI-2 could not be detected in highly invasive breast cancer cell line (MDA-MB-435), while it was expressed in low invasive breast cancer cell lines (MCF-7 and T47D). TFPI-2 mRNA was detected by real-time PCR and the results were corresponded with that of TFPI-2 protein expression (Figure 1b). These data indicated that the expression of TFPI-2 might be regulated at transcriptional level.

### The critical region for TFPI-2 promoter activity in breast cancer cell lines

**Figure 2:**
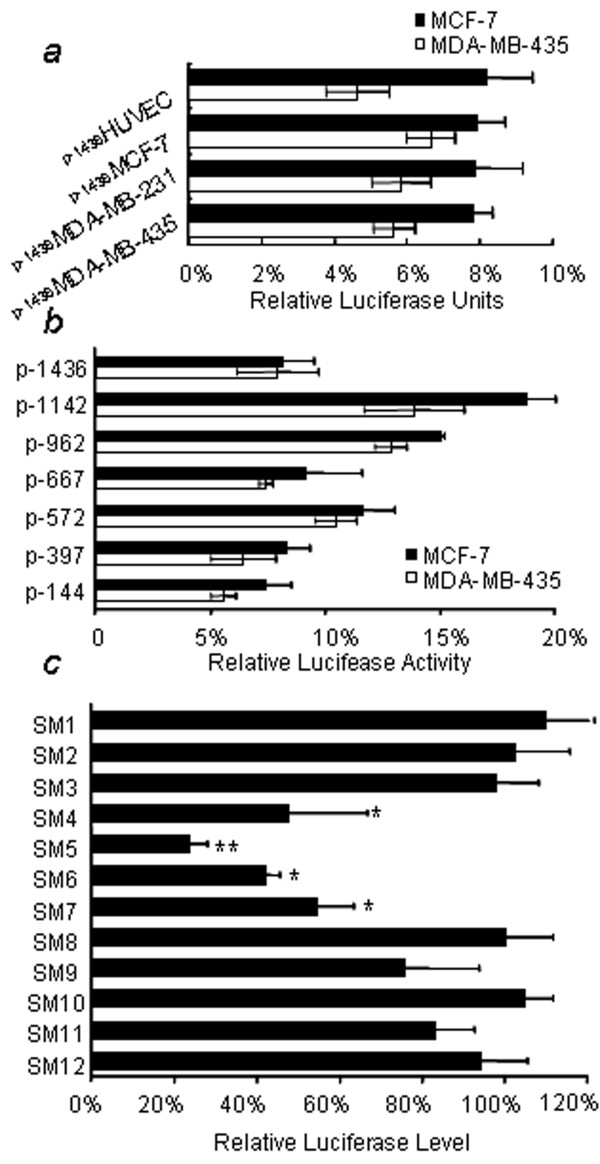
**Analysis of TFPI-2 promoter**. (A) Breast cancer cell lines were transfected with promoter-luciferase plasmid cloned from different cell lines, bearing respective genetic mutation. The promoter from different cell line has nearly the same luciferase activity in both MDA-MB-435 and MCF-7 cell lines. Results are shown as mean ± SD. The apparent lower level of promoter activity in MDA-MB-435 cell was not statistically significant (p = 0.342>0.05). (B) Luciferase reporter gene constructs with 5'ends between nucleotides -1436 and -144 and a common 3'end at +75 were transiently transfected into breast cancer cells. The minimal construct P-144 has the same luciferase activity as P-1436. (C) Linker-scan mutation analysis of TFPI-2 promoter -144 to +1 region. The fragment from -144 to the transcriptional start point (+1) of TFPI-2 promoter were systematically replaced with an BamH I & Xba I polylinker (GGATCCTCTAGA), the resulting sequence were analyzed for not introducing new transcription factor binding site by MatInspector [21]. SM1 indicate the promoter sequence from -12 to +1, and in turn every 12 bases from -144 to -12 was indicated by SM12 to SM2. Then the luciferase promoter reporter plasmids were transfected into MDA-MB-435 cell line and the reported luciferase activity was measured. Results are shown as mean ± SD relative to the wild type (*p < 0.05; **p < 0.001).

To determine if genetic variations contribute to the down regulation of TFPI-2 in breast cancer cells, the potential promoter region (from-1 436 to +75) of TFPI-2 was amplified from various cell lines including normal human umbilical vein endothelial cell (HUVEC), low and high metastatic breast cancer cell lines. After sequencing, several genetic variations were detected among breast cancer cell lines (p-1436MDA-MB-435: -1100 a to g, -1077 a to c, -1072 a to t, -1059 a to g, -876 t to o, -859 t to c, -811 a to g, +13 a to g; p-1436MDA-MB-231: -1222 a to g, -876 t to o, -463 c to t; p-1436MCF-7: -1196 g to c, -735 c to g, -702 c to t, -467 g to c; "o" represents deletion mutation), while the sequence from HUVEC was consistent with what Kamei S had reported (gene bank: AF217542) [14]. Except +13 a to g in MDA-MB-435, all genetic variations lie in the distal region of TFPI-2 promoter. The constructs of TFPI-2 promoter luciferase-reporter bearing respective genetic variations were transiently transfected into breast cancer cells and the luciferase activities were measured according to the manufacture's instruction. As shown in the Figure 2a, the luciferase activities of these constructs showed only slight difference and were not consistent with TFPI-2 expression pattern in these cell lines, indicating that the genetic variations located in the region from -460 to -1142 had no apparent effects on its promoter activities.

To find out the critical region of TFPI-2 promoter in breast cancer cell lines, we constructed several deletion constructs (P-1436, P-1142, P-962, P-667, P-572, P-397 and P-144) and transiently transfected them into MCF-7 cell line. The construct containing -667 to -1142 showed stronger luciferase activity, suggesting the activator/enhancer were presence between -667 to -1142. The transactivation effects were suppressed by the construct containing nucleotides -1142 to -1436, indicating that the repressor/silencer located in this region. In addition, the constructs -144 to -667 exhibited virtually the same luciferase activity as the -1436 construct, suggesting that nucleotides between -144 to +1 contain the sufficient promoter sequence for the expression of human TFPI-2 gene in breast cancer cell (Figure 2b). To further explore the functional relevance of TFPI-2 promoter sequences within -144 to +1, we generated linker-scan mutants in this region. As shown in Figure 2c, the luciferase activities of SM4 to SM7 were obviously decreased (*P *< 0.05), indicating -84 to -36 is critical for TFPI-2 promoter activity. Analysis of this essential region by MatInspector revealed four most possible transcription binding sites including AP1, AP2, KLF6 and SP1 [21]. In addition, the mutation SM5 and SM6 decreased the promoter activity most significantly (*P *< 0.01) and this region contains the core matrix of KLF6, indicating that KLF6 plays an important role in the regulation of TFPI-2 in breast cancer cells.

### KLF 6 can bind and transactivate the promoter of TFPI-2

**Figure 3:**
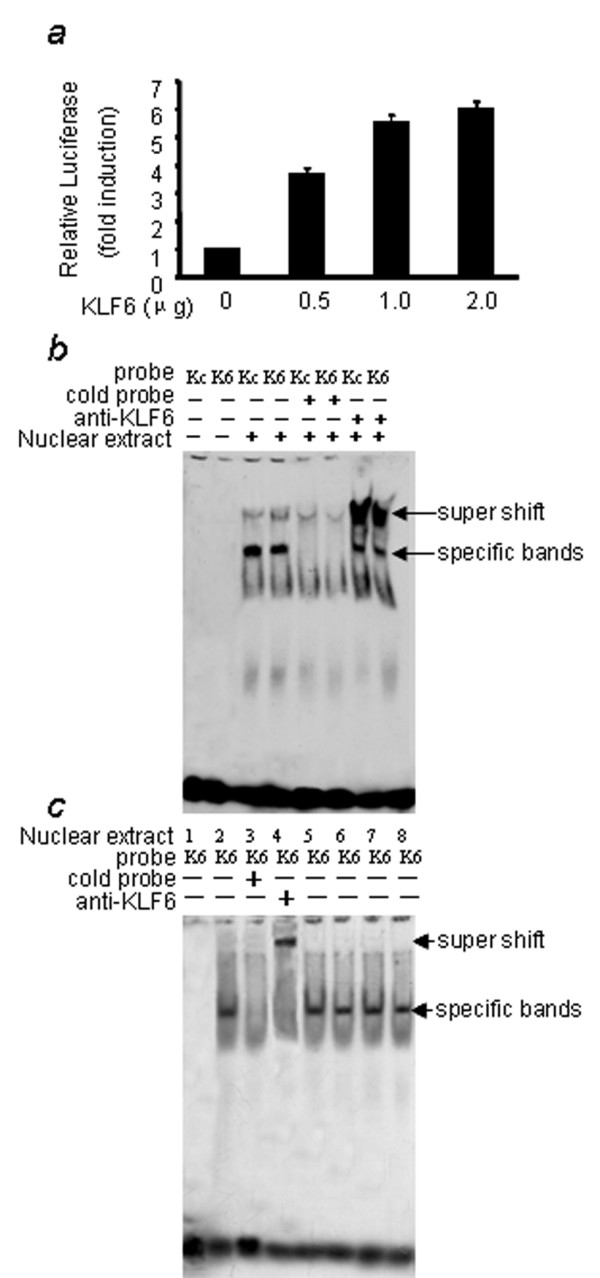
**KLF6 can transactivate and binds to the promoter of TFPI-2**. (A) The p-144 promoter-luciferase activity induced by cotransfection with various amount of KLF6 expression plasmids in MDA-MB-435 cells. The KLF6 cDNA was a generous gift of Scott Friedman and was cloned into PCDNA 3.0 expression vector. (B) Both the predicted KLF6 binding sequence (K6) and the authentic KLF6 binding (Kc) [46] can form specific bands with nuclear extract (NE) from MCF-7 cell lines. And the specific bands were supershifted by the antibody of KLF6. (C) No difference of K6 binding activity was found between MCF-7 and MDA-MB-435 cell lines. Lane 1 to 6: NE from MCF-7; lane 7, 8: NE from MDA-MB-435. Lane 5, 7: 2.5 μg NE; lane 6, 8: 5 μg NE.

We cotransfected p-144 promoter-luciferase constructs with various amounts of KLF6 expression plasmids. As shown in Figure 3a, the transcription activity of p-144 increased 3~5 folder than control accordance with the increasing of KLF6. To further test whether KLF6 binds to -48 ~ -72 region, we performed electrophoretic mobility shift assays (EMSA) using the predicated KLF6 binding site as probe with nuclear extracts prepared from breast cancer cells. As shown in Fig 3b, the probe formed one specific band, consistent with an authentic KLF6 binding motif (CCACCC) and antibodies to KLF6 produced super shifted complexes. There was no difference of KLF6 binding capability between MDA-MB-435 and MCF-7 cell lines (Figure 3c).

### Effects of DNA hypermethylation on the expression of TFPI-2

**Figure 4:**
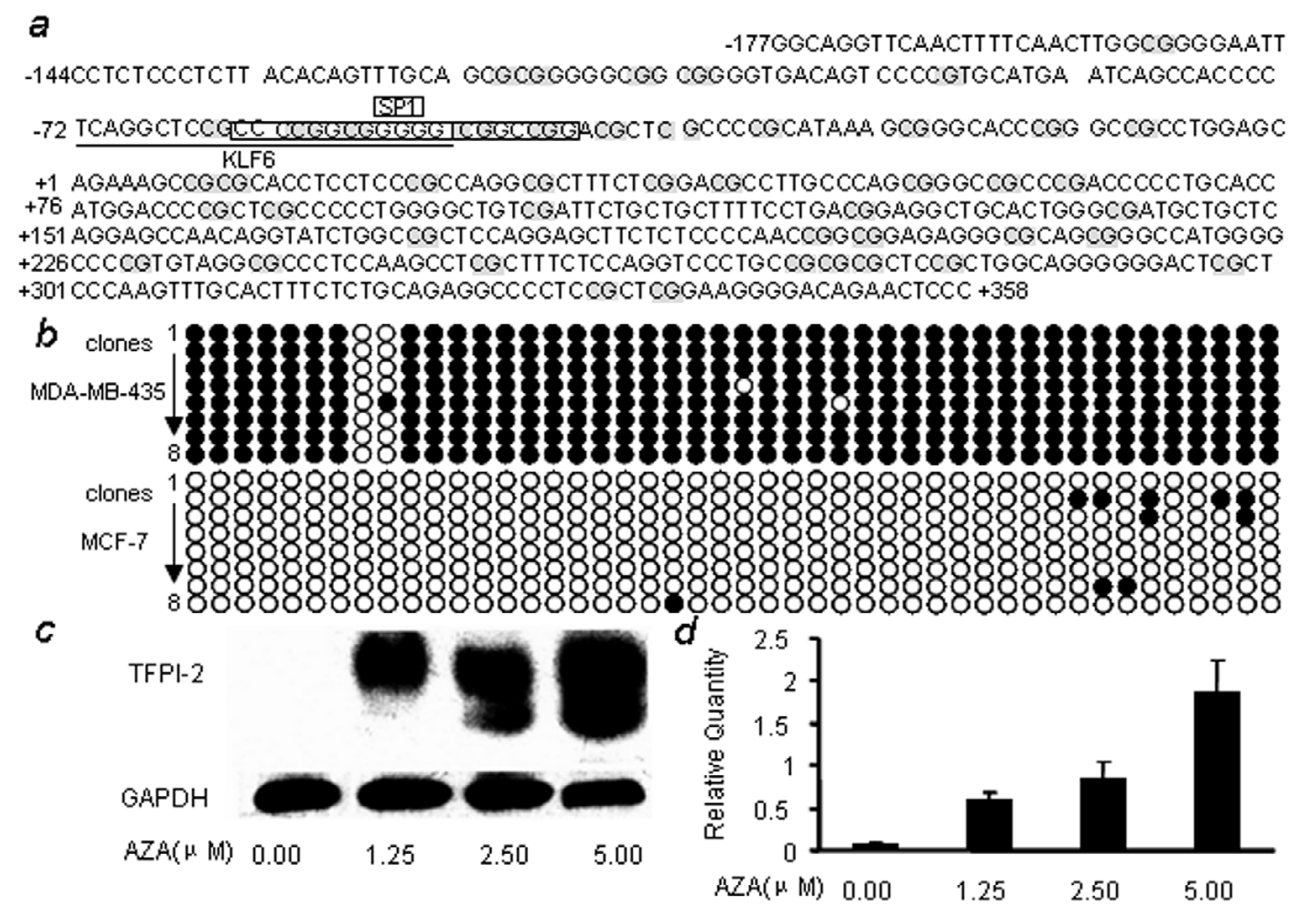
(A) The sequence of CpG islands in TFPI-2 promoter. The CpG dinucleoide detected by bisulfite modified sequence was indicated in gray shadow. (B) The TFPI-2 gene promoter is highly methylated in MDA-MB-435 cells, whereas it is largely unmethylated in MCF-7 cells. For each cell type, the methylation status of 8 individual clones as determined by bisulfite sequencing analyses is shown in rows 1 to 8. The filled or open circles represent the methylated or unmethylated CpG sites, respectively. (C, D) MDA-MB-435 cells were seeded into 10 cm tissue culture dishes at an initial density of 33% confluence, allowed to attach over night, and then replaced with medium containing 1.25, 2.5 and 5 μM of AZA for 72 hour, then expression of TFPI-2 was detected by western-blot (C) and real-time PCR (D).

The 8173 bp DNA fragment ranging from -3 564 to +4 609 of the human TFPI-2 gene was analyzed for CpG islands using online program [22]. After setting the criteria of length >200 nt, with an observed to expected CpG dinucleotide ratio >0.6 and G+C continent >50%, two CpG-rich islands were found (-254 to +374 and +395 to +750). The methylation status of the first CpG islands, encompassing -144 to +1, was determined by bisulfite sequencing. As shown in Figure 4a, all CpG dinucleotides in the region from -177 to +358, except -59 and -56 sites, was methylated in MDA-MB-435 cells, while in MCF-7 cells, no methylation of CpG was. Together with the scan mutation data, we concluded that the binding of KLF6 was affected by CpG methylation, resulting in silence of TFPI-2 in MDA-MB-435 cell line. Treatment of MDA-MB-435 with 5-aza-2'-deoxycitidine, the most commonly used demethylation agent, obviously restored TFPI-2 expression in a concentration-dependent manner (Figure 4b, c), indicating that the down regulation of TFPI-2 in breast cancer cells is related with DNA hypermethylation.

### CpG hypermethylation diminished the binding of KLF6 to TFPI-2 promoter

**Figure 5:**
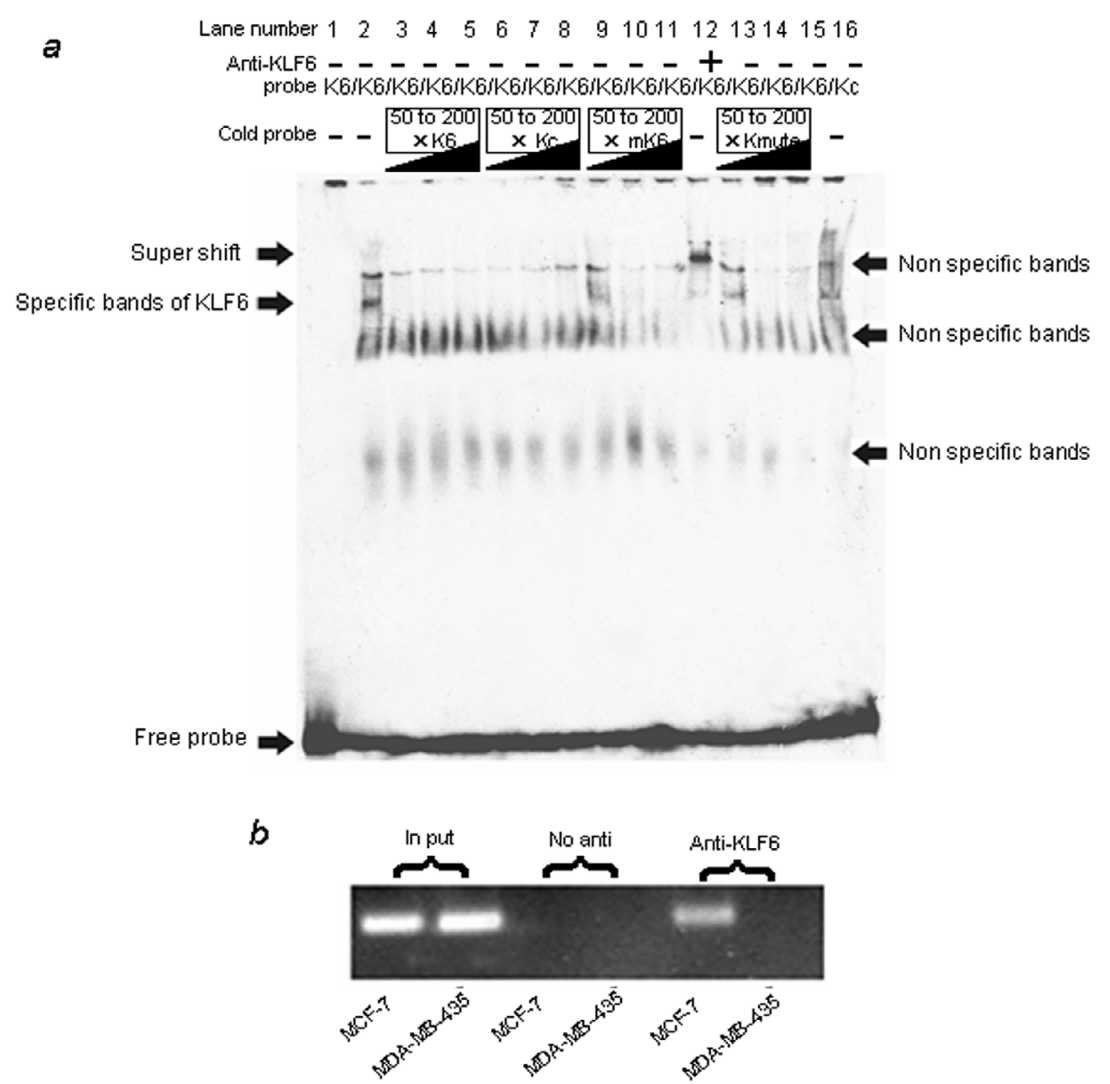
**CpG methylation blocked the binding of KLF6 to TFPI-2 promoter**. (A) CpG methylation in core matrix decreased the binding ability of KLF6 in vitro. Competition were used to determine the affinity of K6, Kc, methylated K6 (mK6) and muted K6 (Kmute). A 50- fold, 100-fold and 200-fold (50×, 100×, 200×) molar excess of cold K6, Kc, mK6 and Kmute cold probe were chased with labeled Kc. All the sequences were listed in table 1 (B) ChIP reveals that hypermethylation blocks KLF6 binding to the TFPI-2 promoter in vivo. Both CpG Unmethylated MCF-7 and methylated MDA-MB-435 cells were tested for binding of KLF6 by ChIP in vivo. Templates for PCR corresponded to the input used in the ChIP assay (input) and DNA obtained from immunoprecipitations performed in the absence (No antibody) or presence of anti KLF6-specific antibody.

To test the effects of CpG methylation on the binding of KLF6, we carried out the competition experiment. In detail, the formation of KLF6 and Kc complex was competed with different concentration (50×, 100×, 200×) of cold Kc, K6, mK6 and Kmute. As shown in Figure 5a, formation of KLF6 complex was abrogated more effectively by competition with K6-50× and Kc-50× than that by competition with mK6-50× and Kmute-50×, indicating the transcription activator KLF6 has higher affinity with unmethylated probe than CpG methylated oligonucleotides, and the nucleotides C in the core matrix of KLF6 binding site was important to KLF6 binding for mutation of C to T decreased the binding affinity obviously. In addition, we used in vivo ChIP assays to demonstrate the effects of CpG methylation on KLF6 binding. KLF6 could bind to TFPI-2 promoter within the CpG islands in MCF-7 cells, for which the TFPI-2 promoter is unmethylated, but not in MDA-MB-435 cells, for which this promoter is methylated (Figure 5b). These data suggested that KLF6 could bind to CpG islands in TFPI-2 promoter and diminished by CpG methylation in its core sequence.

## Discussion

Our study showed that human breast cancer cells exhibited a differential pattern of TFPI-2 expression, with high invasive breast cancer cells (MDA-MB-435) showing no expression of TFPI-2 at all, while much higher levels of TFPI-2 expression were observed in lower invasive MCF-7 and T47D. All three bands reacted with anti-TFPI-2 polyclone antibody were single-gene products with differential glycosylation [23]. It had already been reported that TFPI-2 had an anti-invasive effect that might be mediated via inhibition of plasmin that activates proteases promoting degradation of extracellular matrix and tumor invasion. Several tumor cell lines were less invasive when they were stably transfected with TFPI-2 [5-9]. These results suggest that repression of TFPI-2 gene may be an important event that contributes to breast cancer progression as well as its malignant and invasive phenotype.

Despite the clonal origin of most tumors, their tremendous heterogeneity suggests that cancer progression springs from the combined forces of both genetic and epigenetic events, which produce variant clonal populations, together with the selective pressures of the microenvironment, which promote growth and, perhaps, dissemination of variants with a specific set of characteristics [24]. We hypothesized that both genetic variations and DNA hypermethylation might contribute to the down regulation of TFPI-2 during the progression of breast cancer. To verify this hypothesis, we focused on two cell lines, MCF-7, a hormone-sensitive and low invasive cell line that represents early stage human breast cancer, and MDA-MB-435 cell, which are hormone-insensitive and represent late stage breast cancer [25].

It had already been reported that no sequence variations were detected in any coding exon of TFPI-2 gene in blood donors and apoplectic patients, while the promoter region is relatively polymorphic [26]. We cloned the potential promoter region (from-1 436 to +75) of TFPI-2 gene and found several genetic mutations among breast cancer cell lines. However, these variations located in the region from -1100 to -450 had no apparent effects on their luciferase reporter activities. One possible explanation as why the genetic alteration at a specific locus does not have an impact on its transcriptional activity was the distal region of TFPI-2 promoter where the point mutation located was not critical for TFPI-2 promoter activity [14,27]. The deletion and scan mutation of TFPI-2 promoter support this idea.

Cancers often exhibit aberrant methylations at gene promoter regions that is associated with loss of gene function [28]. TFPI-2 promoter exhibits typical features of a housekeeping gene [8,14]. Analysis with Methprimer revealed that two complete CpG islands spanning promoter and exon 1 and 2 [22]. CpG island hypermethylation has been described in almost every tumor type including breast cancer [13]. It had also been reported that DNMT inhibitor restored expression of TFPI-2 in several cancer cell lines [29-32]. Methylation at the 5' position of cytosine has been reported to alter or interfere with the correct binding of transcription factors to target sequences overlapping CpG dinucleotides [11,12], and it also recruit methyl-CpG binding activities that associate with histone deacetylases and other chromtin-modifying elements that lead to a transcriptionally silenced state.

KLF6, a candidate tumor suppressor gene in prostate cancer [33], contains 283 aa and is present in many tissues, including placenta, heart, lung, liver and pancreas. As an activator of transcription, KLF6 interacts with the core promoter element of a TATA box-less gene [34] and cooperate with other transcription factors in Kruppel-like family, including SP1 and KLF4 [15]. It had already been well documented that the promoter region of TFPI-2 has a high GC-rich content (approximately 75%), with the presence of typical GC boxes known as binding sites for the transcription factor Sp1 [8,35-37]. After we found that there were no difference of KLF6 binding between MDA-MB-435 and MCF-7 cell (Fig 3c), we considered that the cooperation of KLF6 and SP1 might be affected by abnormal hypermethylation in KLF6 binding site.

Treating the cell line MDA-MB-435 with Trichostatin A (TSA) which is a histone deacetylase inhibitor alone (from 100 to 300 ng/ml) could not reactivate expression of TFPI-2, but TSA could enhance the effects of methylase inhibitor AZA (data not shown). The TSA insensitive component of DNMT1 repression may be mediated by its interaction with DNMT associated protein (DMAP1) [38]. These data indicated a cooperative model for histone deacetylation and DNA methylation in maintaining the silence of TFPI-2 in high invasive breast cancer cells. Another question needed to be addressed is why the SP1 binding site in TFPI-2 promoter remains unmethylated in high metastasis breast cancer cell. A possible proposal is that Sp1 elements protect a CpG island from de novo methylation, occurred in tumor genesis [39-41] and this needs to be further investigated.

## Conclusion

In the present work, we shown that: 1) expression of TFPI-2 was repressed in highly invasive breast cancer cell lines; 2) the -84 ~ -36 region was critical for TFPI-2 promoter activity and a KLF6 binding site existed in this region which was abnormally hypermethylated in MDA-MB-435; 3) The CpG methylation in the binding site of KLF-6 blocked the binding of KLF6 to TFPI-2 promoter, diminished the trans-activation of KLF6 on the expression of TFPI-2 gene. On the basis of these findings, we conclude that the down regulation of TFPI-2 in highly metastasis breast cancer was due to the abnormal hypermethylation in CpG dinucleotides of KLF-6 binding site.

## Methods

### Cell cultures and drug treatment

Human breast cancer cell lines MDA-MB-435, MDA-MB-231, T47D and MCF-7 were purchased from American Type Culture collection (ATCC, Manassas, VA), cultured in DMEM with 5% CO_2_. Normal Human Umbilical Vein Endothelial Cell (HUVEC, ATCC, Manassas, VA) were cultured in Ham's F12K supplemented with 30% fetal bovine serum (FBS) and kept at 37°C with 5% CO_2_. All material used in cell culture were purchased from Gibco BRL, with the exception of calf serum which was purchased from PAA Laboratories (Linz, Austria). 5-aza-2'-deoxycitidine (decitabine, AZA) was purchased from Sigma-Aldrich (St Louis, MO, USA)

### Western blot and quantitative real-time PCR analysis

Standard western blot and quantitative real-time PCR analysis were used to analyze protein and RNA expression respectively. Anti-TFPI-2 serum were obtained by immunization of rabbit [42] and anti-GAPDH were purchased from Kangchen (Shanghai, China). Primers targeting TFPI-2 and GAPDH listed in Table 1, were designed using Primer Express software (O-9 to O-12). The PCR reaction was performed in triplicates using the SYBR Green Master Mix (ABI, Warrington, UK) based assay in an ABI 7300 Real Time PCR System according to the manufacturer's instructions.

**Table 1:** Oligonucleotides

List	Sequence
O-1	5'-TCAAGGTACCAGCTTCATACATGCTTGGTTGGGGT-3'
O-2	5'-GTATCTCGAGGGTGCAGGGGGTCGGGCG-3'
O-3	5'-TCGGGGTACCCCTTGGACTACGCAGGAATT-3'
O-4	5'-ACGGGGTACCCCTGGCCTAAGGATGGAAG-3'
O-5	5'-TCGGGGTACCCCATGTTGGCCAGGCTAGTC-3'
O-6	5'-ACGGGGTACCCCCAGCCAAAATGTTCTAATTCTT-3'
O-7	5'-ACGGGGTACCCCATTGCAACGAATCCCG-3'
O-8	5'-TCGGGGTACCCCTCTCCCTCTTACACAGTTTGC-3'
O-9	5' GCTGTGGAGGGAATGACAATAAC 3'
O-10	5' GCGAAGCTTTGGCATCTTCTTT 3'
O-11	5'-GGTGGTCTCCTCTGACTTCAACA-3'
O-12	5'-CAAAGTGGTCGTTGAGGGCA-3'
O-13	5'-GGTTTAATTTTTTAATTTGG-3'
O-14	5'-AAAAATTCTATCCCCTTCC-3'
S1	5'-ACGCTCGCCCCGCATAAAGCGGGCACCCGGGGATCCTCTAGAAGAAAGCCGCGCACCTC-3'
S2	5'-GGGGTCGGCCGGACGCTCGCCCCGCATAAAGGATCCTCTAGAGCCGCCTGGAGCAGAAA-3'
S3	5'-TCCGCCCCGGCGGGGGTCGGCCGGACGCTCGGATCCTCTAGAGCGGGCACCCGGGCCGC-3'
S4	5'-CACCCCTCAGGCTCCGCCCCGGCGGGGGTCGGATCCTCTAGAGCCCCGCATAAAGCGGG-3'
S5	5-'GCATGAATCAGCCACCCCTCAGGCTCCGCCGGATCCTCTAGAGGCCGGACGCTCGCCCC 3'
S6	5'-GACAGTCCCCGTGCATGAATCAGCCACCCCGGATCCTCTAGACCGGCGGGGGTCGGCCG-3'
S7	5'-GGGCGGCGGGGTGACAGTCCCCGTGCATGAGGATCCTCTAGATCAGGCTCCGCCCCGGC-3'
S8	5'-TTTGCAGCGCGGGGGCGGCGGGGTGACAGTGGATCCTCTAGAATCAGCCACCCCTCAGG-3'
S9	5'-CCTCTTACACAGTTTGCAGCGCGGGGGCGGGGATCCTCTAGACCCCGTGCATGAATCAG-3'
S10	5'-GGAATTCCTCTCCCTCTTACACAGTTTGCAGGATCCTCTAGACGGGGTGACAGTCCCCG-3'
S11	5'-TCAACTTGGCGGGGAATTCCTCTCCCTCTTGGATCCTCTAGAGCGCGGGGGCGGCGGGG-3'
S12	5'-AGGTTCAACTTTTCAACTTGGCGGGGAATTGGATCCTCTAGAACACAGTTTGCAGCGCG-3'
KcS	5-GAT CAG GTC ACC CAC AGG CCC-3
KcA	5-GGG CCT GTG GGT GAC CTG ATC-3
K6S	5-CCCTCAGGCTCCGCCCCGGCG-3
K6A	5-CGCCGGGGCGGAGCCTGAGGG-3
mKS	5-CCCTCAGGCTCC(CH_3_)GCCCCGGCG-3
mKA	5-CGCCGGGGC(CH_3_)GGAGCCTGAGGG-3
KmuteA	5-CCCTCAGGCTCTGCCCCGGCG-3
KmuteS	5-CGCCGGGGTGGAGCCTGAGGG-3
C1	5' GTGCATGAATCAGCCACC 3'
C2	5' AGCAGCAGAATCGACAGC 3'

### Amplification of TFPI-2 promoter region and construction of 5' deletion mutants and linker scanning mutants

Genomic DNA was isolated with the genomic DNA isolation kit (Beyotime, Jiangsu, China) according to the manufacture's instruction. A 1.5 Kb fragment of the 5'-flanking region of TFPI-2 was amplified from various cell lines with Super Taq DNA polymerase (Shenergy Biocolor, Shanghai, China) using the oligos O-1 and O-2 to create Kpn I and Xho I restriction site. This Kpn I/Xho I fragment was cloned into the plasmid PGL3-Basic (Promega, Charbonnières, France), named p-1436MDA-MB-435, p-1436MDA-MB-231, p-1436MCF-7 and p-1436HUVEC respectively. The 5' deletion constructs p-1142, p-962, p-667, p-572, p-397 and p-144 were created by PCR using the p-1436 HUVEC as template and O-2, O-3 to O-8 as primers were listed in Table 1. The linker scanning mutants of TFPI-2 promoter were created using PCR-based technique in which consecutive 12-base-pair stretches of wild-type TFPI-2 promoter sequence were replaced with BamH I & Xba I polylinker (GGATCCTCTAGA). This technique has been described previously [43,44]. Primers used were S1 to S12 and O-1, 2. All constructs were confirmed by automated sequencing performed by United Gene Holding, LTD. (Shanghai, China).

### Transient transfection and reporter-luciferase assay

Cells were seeded into six-well plates at a concentration sufficient to give 60–80% confluence on the day of transfection, typically 4 × 10^5 ^cells/well, and cultured overnight. All plasmids for transfection were isolated by QIAGEN plasmid purification kit (QIAGEN, Germany). Transient transfection by jetPEI Polyplus (Poly-plus transfection, France) were performed according to the manufacture's instruction and phRL-CMV Vector (Promega, Charbonnières, France) was used as transfection efficiency control. 48 h after transfection, both firefly and Renilla luciferase activities were measured using Dual-luciferase reporter assay system (Promega, Charbonnières, France) with Luminoskan TL plus Luminometer (Labsystem, Germany) according to the manufacturer's protocol. The relative luciferase units (RLU), which is the ratio of firefly's activity to that of Renilla, were then obtained. Each transfection was carried out in triplicate, and thus the mean and standard deviations were determined.

### Bisulfite modified sequence

Genomic DNA were isolated with the Genomic DNA isolation Kit (Beyotime, Jiangsu, China) and bisulfite treatment was carried out as described [45]. Briefly, genomic DNA was sheared into 200 to 1000 bp fragments by ultrasonication, 2 μg of DNA was diluted into 50 μl, then denaturated for 10 minutes at 37°C with 5.5 μl of 2 M NaOH. Then, 55 μl of freshly prepared hydroxyquinone (10 mM, Sigma-Aldrich) and 520 μl of freshly prepared sodium bisulfite (40.5%, pH = 5, Sigma-Aldrich) were added, mixed, and then incubated under mineral oil at 50°C for 16 hr. Modified DNA was purified using DNA purification resin according to the manufacturer (Shenergy Biocolor, Shanghai, China,) and eluted into 100 μl of water. Modification was completed by NaOH (final concentration, 0.3 M) treatment for 10 min at room temperature, followed by ethanol precipitation. DNA was resuspended in water and used immediately or stored at -20°C. Modified DNA was amplified with FastStarTaq DNA Polymerase (Roche, Mannheim, Germany) by O-13 and O-14. The PCR fragments amplified were gel-purified cloned using pGEM-T vector system (Promega). Eight clones were sequenced to assess the level of methylation in each CpG site.

### Electrophoretic mobility shift assays (EMSA)

Analysis of DNA-protein interactions was performed using DIG Gel Shift Kit (Roche, Mannheim, Germany) according to the manufacture's instructions. Briefly, the double stranded oligonucleotides were 3'-end labeled with DIG-11-ddUTP using terminal transferase. 50 fmol labeled oligonucleotides were incubated with nuclear extract, prepared using cellLytic™ NuCLEAR™ Extraction Kit (Sigma), in binding buffer [20 mM HEPES (pH 7.4), 1 mM MgCl_2_, 10 μM ZnSO_4_, 20 mM KCl, 2% Ficoll, and 1 μg of poly (dI-dC)] for 20 min at 37°C. For competition and supershift assay, the unlabeled oligonucleotides (cold probe) and anti-KLF6 polyclonal antibody (SC-7185x, Santa Cruz, CA, USA) were incubated for 5 min prior to the addition of labeled oligonucleotide. 7% native polyacrylamide gel in 0.5 × TBE (Tris-borate-EDTA buffer) was prepared and used for electrophoresis (in 0.5 × TBE) and gel shift reactions. Blotting was performed using Biorad electro-blotting system according to the manufacturer's instructions. Chemiluminescence detection of DIG-labeled DNA-protein complexes on nylon membranes was detected using GSPD.

### Chromatin Immunoprecipitation (ChIP) assay

ChIP assays were performed using Upstate ChIP Assay Kit (Lake Placid, NY, USA) according to the manufacturer's instructions. In brief, cells were fixed with 1% formaldehyde for 10 min at 37°C. After washing with cold-PBS, cells were lysed in SDS lysis buffer, and sonicated to shear DNA to an average fragment size of 500 bp. Anti-KLF6 antibody (SC-7185x, Santa Cruz, CA, USA) was added. After overnight incubation at 4°C, immune complexes were collected with salmon sperm DNA/protein agarose-50% slurry (Upstate) for 1 h, and then extensively washed. Samples were extracted with elution buffer (1% SDS, 0.1 M NaHCO_3_), and heated at 65°C overnight to reverse crosslinks. DNA was purified and used for PCR. The primer sets (listed in Table 1) were designed to encompass the potential KLF6 binding sites in TFPI-2 gene.

## Abbreviations

TFPI-2: tissue factor pathway inhibitor-2

uPA: urokinasetype plasminogen activator

ECM: extra cellular matrix

KLF6: kruppel-like factor 6

TGF: transforming growth factor

HUVEC: human umbilical vein endothelial cells

EMSA : electrophoretic mobility shift assays

TSA: trichostatin A

DNMT: DNA methyltransferase

DMAP1: DNMT associated protein

ChIP : Chromatin Immunoprecipitation

## Authors' contributions

HG and DM conceived, designed and coordinated the study, and wrote the manuscript. All authors participated in analysis of the results. All authors have read and approved the final manuscript.
